# Professional Courtesy

**Published:** 1887-03

**Authors:** 


### PROFESSIONAL COURTESY.

It would do no member of a dental society any harm carefully to study the address of Dr. J. N. Farrar before the American Academy of Dental Science, as published in our February number. There is no disputing the fact that English societies are conducted in a far more decorous manner than are our own. We sometimes criticise the excessive formality observed at their meetings, but that is infinitely better than the unparliamentary license, the undignified attacks and the unprofessional repartees occasionally indulged in among us. We are earnest in our discussions, but that is no excuse for ungentlemanly conduct. There is no denying that we have professional bullies in some of our societies, who hector and browbeat and insolently treat other members, and too often the listeners, instead of promptly hissing down such swaggerers, and showing their disapprobation of their boorish actions and lack of good breeding, rather applaud the vulgar conduct. It is time that a reform was worked in this matter.

We have more than once seen an honest and well-informed but diffident dentist, who was the guest of a dental society, especially invited to read a paper before it, attacked in the most violent manner, accused of culpable ignorance and lack of common information by some voluble blusterer, in defiance of every principle of hospitality and every sentiment of generosity, and left by the presiding officer and the members to be worried and bull-baited till what of courage he once possessed was completely gone, and he could only vow never again to present a thesis before a society of dentists.

This is all wrong. If a young man — or an old one — presents incorrect theories, or is mistaken in his supposed facts, why is it not as easy to correct him gently? Why should not the decencies and amenities of common society be observed in our meetings at all times? It is not for every one to have an expert knowledge of parliamentary rules, but certainly no dentist who is a gentleman need be totally ignorant concerning—as he certainly will not be entirely regardless of — the commonest instincts of politeness. We have known a member of a society to interrupt another by coarsely, loudly and insolently crying out, "I call the gentleman to order!" and to repeat this whenever the speaker opened his mouth. Such brawling should not be permitted. No one should be so ignorant of parliamentary rules of procedure as not to know that it is only the chairman who can call a member to order. If one believes the rules of order are being transgressed he may "rise to a point of order," and when the chairman calls upon him may "state his point of order," but his words should be addressed to the chair. Indeed, under no circumstances is a member privileged to address any one but the presiding officer when a meeting is in session. This is one of the fundamental laws of procedure, and in English societies it is never disobeyed without rebuke.

We would not be understood as implying that the conduct we have been condemning is common among us, but it is far too frequent when it occurs at all, and hence we are sure that a study of Dr. Farrar's paper would benefit every one. A much greater degree of courtesy in our society meetings would greatly increase their efficiency.

